# Nighttime working as perceived by Italian anesthesiologists: a secondary analysis of an international survey

**DOI:** 10.1186/s44158-023-00119-1

**Published:** 2023-09-11

**Authors:** Alberto Nicolò Galvano, Mariachiara Ippolito, Alberto Noto, Inès Lakbar, Sharon Einav, Antonino Giarratano, Andrea Cortegiani

**Affiliations:** 1 Department of Surgical, Oncological and Oral Science (Di.Chir.On.S.), University of Palermo, Palermo, Italy; 2 Department of Anaesthesia, Intensive Care and Emergency, Policlinico Paolo Giaccone, Via del Vespro 129, 90127 Palermo, Italy; 3 Division of Anesthesia and Intensive Care, Department of Human Pathology of the Adult and Evolutive Age “Gaetano Barresi”, Policlinico “G. Martino,” University of Messina, Messina, Italy; 4 Anesthesiology and Intensive Care, Anesthesia and Critical Care Department B, Saint Eloi Teaching Hospital, PhyMedExp, University of Montpellier, INSERM U1046, 1, 80 Avenue Augustin Fliche, Montpellier Cedex 5, Montpellier, France; 5 General Intensive Care Unit of the Shaare Zedek Medical Centre and the Hebrew University Faculty of Medicine, Jerusalem, Israel

**Keywords:** Nighttime work, Fatigue, Sleep deprivation, Perioperative risk

## Abstract

**Background:**

No data are available on the working conditions and workload of anesthesiologists during perioperative nighttime work in Italy and on the perceived risks.

**Results:**

We analyzed 1085 responses out of the 5292 from the whole dataset. Most of the responders (76%) declared working a median of 12 consecutive hours during night shifts, with an irregular nightshift schedule (70%). More than half of the responders stated to receive a call 2–4 (40%) or 5 times or more (25%) to perform emergency procedures and/or ICU activities during night shifts. More than 70% of the responders declared having relaxation rooms for nighttime work (74%) but none to be used after a nightshift before going back home (82%) and no free meals, snacks, or beverages (89%). Furthermore, almost all (95%) of the surveyed anesthesiologists declared not having received specifical training or education on how to work at night, and that no institutional program has been held by the hospital to monitor fatigue or stress for night workers (99%). More than half of the responders stated having the possibility, sometimes (38%) or always (45%), to involve another colleague in difficult medical decisions and to feel comfortable, sometimes (31%) or always (35%), to call the on-call colleague. Participants declared that nighttime work affects their quality of life extremely (14%) or significantly (63%), and that sleep deprivation, fatigue, and current working conditions may reduce performance (67%) and increase risk for the patients (74%).

**Conclusions:**

Italian anesthesiologists declare current nighttime practice to negatively affect their quality of life, and their performance, and are thus concerned for their patients’ safety. Proper education on night work, starting from traineeship, and implementing institutional programs to monitor stress and fatigue of operators and to support them during nighttime work could be a mean to improve nighttime work conditions and safety for both patients and healthcare workers.

**Supplementary Information:**

The online version contains supplementary material available at 10.1186/s44158-023-00119-1.

## Background

The risks and poor conditions of nighttime perioperative work have been recently highlighted by several studies [1–16] and received increasing scientific attention. Recently, an international survey has been conducted investigating the burden of perioperative work at night as perceived by anesthesiologists, collecting responses from more than 5000 participants who perceive current practice as adversely affecting their professional performance and the safety of their patients [17]. A significant impact was also reported on anesthesiologists’ own quality of life [17]. Despite common themes being present across different countries, variability is reasonable due to different regulations, healthcare systems, work characteristics, and available resources. In Italy, the shift schedule in public hospitals, usually organized according to the hospital service needs, must respect the National Collective Agreement of Work [18]. It states that a medical doctor’s weekly workload is of 38 h, with an 11-h period of rest after 12 consecutive hours of work. The same hour limit applies to the private setting, which is regulated by the National Collective Agreement of Work of private hospitals [19]. In terms of occupational medicine and prevention programs, Italy has recently identified doctors working nightshifts as workers with right to early retirement, based on the number of years of nighttime working [20]. All these characteristics may distinguish the impact of nighttime work on anesthesiologists working in Italy from that of those working elsewhere. The aim of this secondary analysis of an international survey of anesthesiologists was to specifically address the Italian working conditions and their impact on patients’ safety and healthcare workers’ wellbeing.

## Methods

A secondary analysis was conducted on the data provided by the respondents who participated in an international survey and working in Italy as anesthesiologists. The complete description of the survey instrument and administration methods is available in the report of the primary analysis [17]. In brief, the survey was administered through a web-based platform, and it consisted of 28 closed questions. An introductory validation question asked the respondents to attest they were currently involved in the perioperative care as anesthesiologists. As a review step, the platform asked to confirm the answer, before submitting. The investigated topics were as follows: (1) demographic characteristics of the respondents and of the hospital they work in (academic or nonacademic, public or private), (2) nightshift schedule strategy and nighttime workload, in the perioperative setting and/or in ICUs, (3) availability of rest facilities and advocacy during nightshifts, and (4) perceived impact of nighttime working conditions on patients’ safety, professionals’ quality of life, and their performance. Data cleaning was already performed for the original study, and, for the purpose of this secondary analysis, a cleaned version of the main database was used. Data were then extracted and filtered selecting “Italy” as working country. Data analysis was then conducted using descriptive statistics and presented using including frequencies and percentages or median and interquartile range as appropriate. Subgroup analyses were also conducted on responses obtained from (1) trainees, (2) professionals declaring being called almost every time when on-call, and (3) anesthesiologists declaring that nighttime work affects, significantly or extremely, their quality of daily life. These analyses are available as tables in the Additional file 1. Data analysis and graphical presentation were performed by ANG with input by MI, AN, SE, and AC using Microsoft Excel (version 16.73 Microsoft Office365; Microsoft Corporation, Redmond, CA, USA).

## Results

A total of 1085 responses were extracted from the database as provided by professionals working in Italy. The characteristics of the respondents are shown in Table 1. A prevalence of female responders has emerged (56%). Around 76% of the responders declared an age between 30–55 years. Most of responders declared working in public institutions (92%), 47% in academic hospitals.

**Table 1:** Demographic characteristics of the respondents

		**All (*n* = 1085)**
		*N* (%)
**Please indicate your age**	< *30 years old*	87 (8%)
	> *55 years old*	167 (16%)
	*30–40 years old*	436 (40%)
	*41–55 years old*	395 (36%)
**Please indicate your status**	*Junior consultant (*< *10 years since passing boards)*	383 (35%)
	*Senior consultant*	545 (50%)
	*Trainee*	157 (15%)
**Please indicate to which gender identity do you most identify**	*Female*	610 (56%)
	*Male*	475 (44%)
**Is the hospital where you work as follows?**	*Academic*	511 (47%)
	*Nonacademic*	574 (53%)
**Is the hospital where you work as follows?**	*Private*	87 (8%)
	*Public*	998 (92%)

Data are reported as number and percentages

### Nighttime workload

Table 2 shows the responses to the survey section exploring the aspects of the respondents’ anesthesia practice.

**Table 2:** Nighttime workload characteristics of the respondents

		**All (*n* = 1085)**
		N (%)
**How many nights are you on-call (standby at home) every month?**		3 [1–4]
**On nights that you are on call (standby at home), how many times you get called into the hospital on an average?**	*I’m not on call (at home)*	252 (23%)
	*Almost every time*	130 (12%)
	*Occasionally*	396 (37%)
	*Rarely*	307 (28%)
***How many nights do you work on site (all night in the hospital) every month?***		4 [4, 5]
**How many times are you called per night when you are on site shift (all night in the in-hospital)?**	*I’m not on site*	16 (2%)
	*2–4 times*	438 (40%)
	*Five times or more*	270 (25%)
	*One time a night at most*	66 (6%)
	*We work all night running*	295 (27%)
**When you work during the night, how many consecutive work hours does your shift include overall?**	*I’m not on site*	16 (1%)
	*6*	120 (11%)
	*8*	73 (7%)
	*12*	816 (76%)
	*16*	25 (2%)
	*24*	19 (2%)
	*Other*	16 (1%)
**Which kind of schedule strategy is employed in your hospital?**	*Consecutive night shifts (number of night shifts in a row alternating with number of day shifts in a row)*	25 (2%)
	*Irregular night shifts*	760 (70%)
	*Permanent night shift work (primarily or only night shifts)*	45 (4%)
	*Rotating shift with 1 day off (night shift and day shift together and then 1 recovery day)*	210 (20%)
	*Rotating shift with 2 days off (night shift and day shift together and then 2 recovery days)*	45 (4%)
**Is it common for you to work at night after having worked on the same day?**	*No, never*	557 (51%)
	*Rarely*	275 (25%)
	*Sometimes*	137 (13%)
	*Yes, frequently*	76 (7%)
	*Yes, always*	40 (4%)
**What type of patient do you attend to during nighttime?**	*Postanesthesia care unit/intensive care unit team activity*	676 (62%)
	*Emergency surgery*	815 (75%)
	*Elective Surgery*	104 (10%)
	*Emergency medical team activity*	551 (51%)

Data are reported as number and percentages or median and interquartile range as appropriate

Professionals declared working a median of 4 [IQR 4 to 5] on-site and 3 [*IQR* 1 to 4] on-call night shifts per month with mostly irregular night shifts as schedule strategy (70%). Nightshift durations were reported to be usually 12 h (76%), with 2% declaring a duration of 24 h. More than 10% of the respondents declared frequently or always working at night after having worked during the day, while another 25% declared it to occur on rare occasions. In the subgroup analysis of the respondents declaring being called at work almost every time when on-call shift, 29% of them stated having to work the day after, frequently (19%) or always (10%) (Table S10. Additional file 1).

During on-site night shifts, most responders declared receiving 2–4 (40%) or 5 times or more (25%) requests to perform emergency surgery (75%), intensive care unit activities (62%), and/or emergency medical team activities (51%).

### Facilities and advocacy

The second part of the survey inquired about facilities available during night shifts and the hospital activities aimed to mitigate risks related to nighttime work. Full responses to this section are presented in Table 3.

**Table 3:** Results of questions on facilities and advocacy

		**All (*n* = 1085)**
		*N* (%)
**Does your hospital have rooms dedicated to relaxation for all doctors working at night?**	*No*	286 (26%)
	*Yes*	799 (74%)
**Does your hospital have rest facilities available for doctors who have worked during the night, to be used before returning home?**	*No*	893 (82%)
	*Yes*	192 (18%)
**Does your hospital provide free meals, snacks, and beverages (i.e., water, coffee, tea) to doctors working at night?**	*No*	965 (89%)
	*Yes*	120 (11%)
**Were you informed of the consequences of nightwork before you started working as a trainee?**	*No*	860 (79%)
	*Yes*	225 (21%)
**Have you ever received training, information, or tips on how to improve your performance when working at night?**	*No*	1034 (95%)
	*Yes*	51 (5%)
**Does your hospital have a program to monitor stress or fatigue in night shift workers?**	*No*	1075 (99%)
	*Yes*	10 (1%)
**During nighttime, do you have the possibility to discuss clinical issues or involve another colleague in difficult clinical decisions?**	*Usually, no*	183 (17%)
	*Sometimes*	418 (38%)
	*Yes, always when I feel I need it*	484 (45%)
**During nighttime, do you feel comfortable calling the person “on call” to come into the hospital?**	*No*	374 (34%)
	*Sometimes*	332 (31%)
	*Yes*	379 (35%)
**You responded “only sometimes” or “no” please state the MAIN cause** **Total = 706**	*Other*	39 (5%)
	*The person I am calling in is senior to me*	61 (9%)
	*The person I am calling in may argue with me about the need to come on the basis of case complexity or the number of cases*	146 (21%)
	*The person I am calling in or my colleagues may think I am not suited for this job*	29 (4%)
	*The person I am calling in will be calling me in on another day*	39 (6%)
	*The person I am calling in will be judging my performance when my tenure/promotion is brought up for discussion*	24 (3%)
	*The person I am calling works on the following day, and I do not want to deprive them of sleep*	368 (52%)

Data are reported as number and percentages

A high percentage of the respondents (79%) were not informed of the consequences of nightwork before they started working as trainees, and almost all of them (95%) had never received training or information on how to improve their performance when working at night. Almost all (99%) the respondents declared their centers have no institutional programs to monitor stress or fatigue among nighttime workers.

Approximately, 80% of the respondents (45% “yes, always when I feel I need it,” 38% “sometimes”) stated they have the possibility to discuss clinical issues or involve another colleague in difficult clinical decisions during nighttime, and more than half (66%) of them feel comfortable, sometimes or always, calling the person “on-call” to come into the hospital for help.

### Perceived impact of nighttime work

The perceptions of the respondents on the effects of nighttime work were evaluated on two separate topics: their own quality of life (one question) and the safety of their patients (four questions).

In response to the question addressing quality of life, 77% of the respondents declared being negatively affected by nighttime work, with 14% of them declaring it is extremely affecting. Analyzing the trainees subgroup only, 65% of them declared their quality of daily life is affected significantly (56%) or extremely (9%) (Table S4. Additional file 1).

Considering the questions on patient safety, more than half of respondents declared believing that sleep deprivation negatively affects their professional performance extremely or significantly (67%), and that their fatigue during nighttime work may increase the perioperative risk of their patients to some degree or very much (65%); most also agreed or strongly agreed with the phrase “Night-time work represents an additional risk per se for the patient” (77%), and most of them declared that the overall working conditions at their hospital during nighttime may increase partly or very much the perioperative risk of their patients (74%). The full responses to this section of the survey are shown in Table 4.

**Table 4:** Results of questions on patients’ safety and doctors’ quality of life

		**All (*n* = 1085)**
		*N* (%)
**Please indicate how much you think your nightwork affects the quality of your daily life**	*Extremely*	161 (14%)
	*Significantly*	679 (63%)
	*Neutral*	119 (11%)
	*Slightly*	119 (11%)
	*Not at all*	7 (1%)
**Do you believe that sleep deprivation affects your professional performance?**	*Extremely*	143 (13%)
	*Significantly*	584 (54%)
	*Neutral*	164 (15%)
	*Slightly*	163 (15%)
	*Not at all*	31 (3%)
**Do you believe that your fatigue during nighttime work may increase the perioperative risk of your patients?**	*Very much*	170 (16%)
	*To some degree*	531 (49%)
	*Neutral*	158 (14%)
	*Rarely*	170 (16%)
	*Not at all*	56 (5%)
**Taking into account your current work conditions, please rate your opinion on the following sentence: “Night-time work represents an additional risk per se for the patient”**	*Strongly agree*	234 (22%)
	*Agree*	600 (55%)
	*Neutral*	174 (16%)
	*Disagree*	66 (6%)
	*Strongly disagree*	11 (1%)
**Do you believe that the overall working conditions at your hospital during nighttime may increase the perioperative risk of your patients?**	*Very much*	226 (21%)
	*Partly*	568 (53%)
	*Neutral*	144 (13%)
	*Rarely*	112 (10%)
	*Not at all*	35 (3%)

Data are reported as number and percentages

In the subgroup analysis of those declaring nighttime work negatively affects, significantly or extremely, their quality of daily life; 76% declared that sleep deprivation may affect their performance (Table S8. Additional file 1).

## Discussion

In this secondary analysis of an international survey, 1085 responses by anesthesiologists working in Italy have been analyzed. More than half of the respondents surveyed believe their nighttime working condition may increase the perioperative risk of their patients, and that it has a negative impact on their quality of life. They reported having rest facilities available during night shift but none to be used after, and their hospitals do not provide free water or meals during nighttime work. They also declare to have never received any training on nighttime work, and that programs to monitor stress or fatigue are lacking. Our findings are mostly in line with the results of the main study, offering a picture of the Italian situation and contributing to the interpretation of the independent higher risk of morbidity and mortality observed among patients undergoing nighttime surgery, which is not fully explained by measurable surgical or clinical factors [21–23]. However, considering the high proportion of the respondents in the main survey declared working in Italy (20%), some of the results coming from this secondary analysis are relevant and may be considered as important feedback on potential area of improvement in the field of anesthesia and intensive care in Italy. Awareness of the current conditions of nighttime work should bring both individuals and institutions to implement changes. The most strikingly negative finding was that 11% of the respondents declared frequently or always working at night after having worked during the day. Indeed, despite such practice is currently forbidden by law, it is probably still performed in centers, especially where shortage of personal is more relevant. The picture is completed by the absence of educational training and institutional programs to monitor stress and fatigue of healthcare workers. It is demonstrated that fatigue and sleep deprivation that occur during night shifts reduce critical thinking and ability to take appropriate complex decision, especially in the emergency setting such as the one anesthesiologists work in [10, 12]. Moreover, well-being of the operators can be heavily affected by sleep deprivation [9]. In our opinion, this could partially contribute to the lack of anesthesiologists that Italian healthcare system is suffering. At the same time, also other healthcare specialties are facing nowadays an increasing shortage in numbers of physicians [24]. During nighttime, the poor number of expert physicians on site may lead to an increase in requests for anesthesiology consultations to help other medical specialties in case of emergencies. This may contribute to developing stress and increase the risk of burnout [13]. Open discussion of clinical cases among colleagues and/or shared decisions on clinical issues may be another countermeasure to approach the problem. The Italian situation, in this case, seems not dramatic: almost half of the respondents (45%) declared always having the possibility to discuss clinical cases with another colleague if needed. Nonetheless, 17% of the surveyed professionals declared not having this opportunity, while 38% stated having it but “sometimes.” Improving this issue may be effective to reduce negative impact of night work.

We acknowledge that different seniority of anesthesiologists may be associated with different perception of impact of nighttime work on both professional and personal life due to the specific physical and mental abilities and different lifestyles. On the other hand, longer professional experience may also lead to enhanced ability to manage the challenge of nighttime work in comparison with the early stage of the career. Thus, we performed a subgroup analysis including the response from trainees, which resulted in line with data from the entire Italian cohort. Moreover, our data showed that respondents declaring nightwork affects, significantly or extremely, their quality of daily life have similar age distribution in comparison to the whole cohort (Table S5. Additional file 1).

Specifically looking at the respondents declaring nightwork affects their quality of daily life significantly or extremely (77%), we can speculate that those who are more burdened by nightwork seem also to be more heavily worried of their functioning at work due to sleep deprivation, as they believe that it affects, extremely (16%) or significantly (60%), their professional performance. However, these exploratory analyses were performed as post hoc subgroup analyses and should be considered as hypothesis generating. Our study also collected data on countermeasures, in terms of knowledge and training on the topic.

The majority of respondents declared to have never received training on how to face nightwork, and Italian institutions did not provide specific guidelines or fatigue monitoring programs. Some of the most important measures, such as reducing work hours and/or implementing the number of healthcare workers, may be feasible but with costs. Other measures may be more easily and rapidly implemented, such as availability of facilities [25]. In addition, the use of simulation and training programs may provide support and competencies for coping the burden of nightshifts working. A recent observational study conducted by Couarraze et al. [26] showed that simulation training may be effective in reducing perceived stress and, thus, the risk of burnout. Other centers have implemented fatigue risk management systems [27, 28]. As an example, a review by Sprajcer et al. [29], which analyzed different fatigue risk management systems in different settings and countries, has concluded that these may improve safety outcomes on fatigue-related complications. Positive experiences could be considered as background to build evidence and guide regulations and guidelines on the topic.

Overall, the study should be considered along with its limitations. First, as per the main study, no reliable response rate can be estimated, due to the multiple ways the survey was distributed across the country. Self-selection bias may also be an issue, as more respondents may have been professionals more sensible to the topic and/or working in particularly unfavorable or favorable conditions. This limitation is partly confirmed by the almost lack of responses from private hospitals, which are widely present in the country, and that may apply different schedule policies compared to public ones. Furthermore, we did not specifically investigate the type of employment of the respondents in terms of working hours. We also did not collect data to compare respondents only working on-call during night with those only working on-site. Finally, no data were collected on patients’ outcome. However, strengths should also be considered, as the study provides the first available data on the topic in Italy, with a reasonably large number of responses. These results, together with the growing evidence on patient safety and healthcare workers wellbeing, may provide useful insights to improve the working condition in Italy.

## Conclusions

Even though Italian law provides some regulation on medical working, Italian anesthesiologists still declare current nighttime practice to negatively affect their quality of life and their performance and are thus concerned for their patients’ safety. Proper education on night work, starting from traineeship, and implementing institutional programs to monitor stress and fatigue of operators and to support them during nighttime work could be a mean to improve nighttime work conditions for both patients and healthcare workers.

### Supplementary Information

**Additional file 1:** **Table S1.** Demographic characteristics of the respondents declaring being a trainee. **Table S2.** Nighttime workload characteristics of the respondents declaring being a trainee. **Table S3.** Results of questions on facilities and advocacy of the respondents declaring being a trainee. **Table S4.** Results of questions on patients’ safety and doctors’ quality of life of the respondents declaring being a trainee. **Table S5.** Demographic characteristics of the respondents declaring nightwork affects, significantly or extremely, their quality of daily life. **Table S6.** Nighttime workload characteristics of the respondents declaring nightwork affects, significantly or extremely, their quality of daily life. **Table S7.** Results of questions on facilities and advocacy of the respondents declaring nightwork affects, significantly or extremely, their quality of daily life. **Table S8.** Results of questions on patients’ safety and doctors’ quality of life of the respondents declaring nightwork affects, significantly or extremely, their quality of daily life. **Table S9.** Demographic characteristics of the respondents declaring being called at work almost every time when on-call. **Table S10.** Nighttime workload characteristics of the respondents declaring being called at work almost every time when on-call. **Table S11.** Results of questions on facilities and advocacy of the respondents declaring being called at work almost every time when on-call. **Table S12.** Results of questions on patients’ safety and doctors’ quality of life of the respondents declaring being called at work almost every time when on-call.

## Data Availability

The data that supports the findings of this study are available from the corresponding author, upon reasonable request.
