# Supplementary material for: Nighttime working as perceived by Italian anesthesiologists: a secondary analysis of an international survey
Source: J Anesth Analg Crit Care. 2023 Sep 11;3:32. doi: 10.1186/s44158-023-00119-1 (PMC10494393; doi:10.1186/s44158-023-00119-1)
Supplement: Supplementary file 1 — Additional file 1: Table S1. Demographic characteristics of the respondents declaring being a trainee. Table S2. Nighttime workload characteristics of the respondents declaring being a trainee. Table S3. Results of questions on facilities and advocacy of the respondents declaring being a trainee. Table S4. Results of questions on patients’ safety and doctors’ quality of life of the respondents declaring being a trainee. Table S5. Demographic characteristics of the respondents declaring nightwork affects, significantly or extremely, their quality of daily life. Table S6. Nighttime workload characteristics of the respondents declaring nightwork affects, significantly or extremely, their quality of daily life. Table S7. Results of questions on facilities and advocacy of the respondents declaring nightwork affects, significantly or extremely, their quality of daily life. Table S8. Results of questions on patients’ safety and doctors’ quality of life of the respondents declaring nightwork affects, significantly or extremely, their quality of daily life. Table S9. Demographic characteristics of the respondents declaring being called at work almost every time when on-call. Table S10. Nighttime workload characteristics of the respondents declaring being called at work almost every time when on-call. Table S11. Results of questions on facilities and advocacy of the respondents declaring being called at work almost every time when on-call. Table S12. Results of questions on patients’ safety and doctors’ quality of life of the respondents declaring being called at work almost every time when on-call. [file 44158_2023_119_MOESM1_ESM.docx]

Supplementary material to:

**Nighttime working as perceived by Italian anesthesiologists:**

**A secondary analysis of an international survey**

**List of Contents**

**Table S1. Demographic characteristics of the respondents declaring being a trainee.**

**Table S2. Nighttime workload characteristics of the respondents declaring being a trainee.**

**Table S3. Results of questions on facilities and advocacy of the respondents declaring being a trainee.**

**Table S4. Results of questions on patients’ safety and doctors’ quality of life of the respondents declaring being a trainee.**

**Table S5. Demographic characteristics of the respondents declaring nightwork affects, significantly or extremely, their quality of daily life.**

**Table S6. Nighttime workload characteristics of the respondents declaring nightwork affects, significantly or extremely, their quality of daily life.**

**Table S7. Results of questions on facilities and advocacy of the respondents declaring nightwork affects, significantly or extremely, their quality of daily life.**

**Table S8. Results of questions on patients’ safety and doctors’ quality of life of the respondents declaring nightwork affects, significantly or extremely, their quality of daily life.**

**Table S9. Demographic characteristics of the respondents declaring being called at work almost every time when on-call.**

**Table S10. Nighttime workload characteristics of the respondents declaring being called at work almost every time when on-call.**

**Table S11. Results of questions on facilities and advocacy of the respondents declaring being called at work almost every time when on-call.**

**Table S12. Results of questions on patients’ safety and doctors’ quality of life of the respondents declaring being called at work almost every time when on-call.**

**Table S1. Demographic characteristics of the respondents declaring being a trainee.**

|  | | | Trainees  (n=157) | |
| --- | --- | --- | --- | --- |
|  | | | N (%) | |
| **Please indicate your age** | *< 30 years old* | 77 (49%) | |  |
|  | *> 55 years old* | 3 (2%) | |  |
|  | *30-40 years old* | 74 (47%) | |  |
|  | *41-55 years old* | 3 (2%) | |  |
| **Please indicate your status** | *Junior consultant (<10 years since passing boards)* | 0 (0%) | |  |
|  | *Senior consultant* | 0 (0%) | |  |
|  | *Trainee* | 157 (100%) | |  |
| **Please indicate to which gender identity do you most identify?** | *Female* | 94 (60%) | |  |
|  | *Male* | 63 (40%) | |  |
| **Is the hospital where you work:** | *Academic* | 144 (92%) | |  |
|  | *Non academic* | 13 (8%) | |  |
| **Is the hospital where you work:** | *Private* | 2 (1%) | |  |
|  | *Public* | 155 (99%) | |  |

Data are reported as number and percentages.

**Table S2. Nighttime workload characteristics of the respondents declaring being a trainee.**

|  | | Trainees  (n=157) |
| --- | --- | --- |
|  | | N (%) |
| **How many nights are you on-call (standby at home) every month?** | | 0 [0-0]* |
| **On nights that you are on call (standby at home), how many times you get called into the hospital on an average?** | *I’m not on call (at home)* | 119 (76%) |
|  | *Almost every time* | 9 (6%) |
|  | *Occasionally* | 19 (12%) |
|  | *Rarely* | 10 (6%) |
| ***How many nights do you work on site (all night in the hospital) every month?*** | | 5 [4-6] |
| **How many times are you called per night when you are on site shift (all night in the in-hospital)?** | *I’m not on site* | 2 (1%) |
|  | *2-4 times* | 79 (50%) |
|  | *Five times or more* | 32 (21%) |
|  | *One time a night at most* | 9 (6%) |
|  | *We work all night running* | 35 (22%) |
| **When you work during the night, how many consecutive work hours does your shift include overall?** | *I’m not on site* | 2 (1%) |
|  | *6* | 17 (11%) |
|  | *8* | 9 (6%) |
|  | *12* | 120 (77%) |
|  | *16* | 5 (3%) |
|  | *24* | 2 (1%) |
|  | *Other* | 2 (1%) |
| **Which kind of schedule strategy is employed in your hospital?** | *Consecutive night shifts (number of night shifts in a row alternating with number of day shifts in a row)* | 4 (3%) |
|  | *Irregular night shifts* | 84 (53%) |
|  | *Permanent night shift work (primarily or only night shifts)* | 5 (3%) |
|  | *Rotating shift with 1 day off (night shift and day shift together and then 1 recovery day)* | 49 (31%) |
|  | *Rotating shift with 2 days off (night shift and day shift together and then 2 recovery days)* | 15 (10%) |
| **Is it common for you to work at night after having worked on the same day?** | *No, never* | 89 (57%) |
|  | *Rarely* | 37 (24%) |
|  | *Sometimes* | 19 (12%) |
|  | *Yes, frequently* | 10 (6%) |
|  | *Yes, always* | 2 (1%) |
| **What type of patient do you attend to during night-time?** | *Post Anesthesia Care Unit / Intensive Care Unit team activity* | 107 (68%) |
|  | *Emergency surgery* | 119 (76%) |
|  | *Elective Surgery* | 5 (3%) |
|  | *Emergency medical team activity* | 91 (58%) |

Data are reported as number and percentages.

*Range min-max [0-24]

**Table S3. Results of questions on facilities and advocacy of the respondents declaring being a trainee.**

|  | | Trainees  (n=157) |
| --- | --- | --- |
|  | | N (%) |
| **Does your hospital have rooms dedicated to relaxation for all doctors working at night?** | *No* | 54 (34%) |
|  | *Yes* | 103 (66%) |
| **Does your hospital have rest facilities available for doctors who have worked during the night, to be used before returning home?** | *No* | 133 (85%) |
|  | *Yes* | 24 (15%) |
| **Does your hospital provide free meals, snacks, and beverages (i.e. water, coffee, tea) to doctors working at night?** | *No* | 148 (94%) |
|  | *Yes* | 9 (6%) |
| **Were you informed of the consequences of nightwork before you started working as a trainee?** | *No* | 117 (74%) |
|  | *Yes* | 40 (26%) |
| **Have you ever received training, information or tips on how to improve your performance when working at night?** | *No* | 143 (91%) |
|  | *Yes* | 14 (9%) |
| **Does your hospital have a program to monitor stress or fatigue in night shift workers?** | *No* | 156 (99%) |
|  | *Yes* | 1 (1%) |
| **During night-time, do you have the possibility to discuss clinical issues or involve another colleague in difficult clinical decisions?** | *Usually, no* | 13 (8%) |
|  | *Sometimes* | 78 (50%) |
|  | *Yes, always when I feel I need it* | 66 (42%) |
| **During night-time, do you feel comfortable calling the person 'on call' to come into the hospital?** | *No* | 51 (33%) |
|  | *Sometimes* | 60 (38%) |
|  | *Yes* | 46 (29%) |
| **You responded 'only sometimes' or 'no' please state the MAIN cause:**  **(Total=109)** | *Other* | 6 (5%) |
|  | *The person I am calling in is senior to me* | 22 (20%) |
|  | *The person I am calling in may argue with me about the need to come on the basis of case complexity or the number of cases* | 27 (24%) |
|  | *The person I am calling in or my colleagues may think I am not suited for this job* | 5 (5%) |
|  | *The person I am calling in will be calling me in on another day.* | 4 (4%) |
|  | *The person I am calling in will be judging my performance when my tenure/promotion is brought up for discussion* | 6 (5%) |
|  | *The person I am calling works on the following day and I do not want to deprive them of sleep.* | 41 (37%) |

Data are reported as number and percentages.

**Table S4. Results of questions on patients’ safety and doctors’ quality of life of the respondents declaring being a trainee.**

|  |  | Trainees  (n=157) |
| --- | --- | --- |
|  |  | N (%) |
| **Please indicate how much you think your nightwork affects the quality of your daily life** | *Extremely* | 14 (9%) |
|  | *Significantly* | 88 (56%) |
|  | *Neutral* | 32 (20%) |
|  | *Slightly* | 22 (14%) |
|  | *Not at all* | 1 (1%) |
| **Do you believe that sleep deprivation affects your professional performance?** | *Extremely* | 18 (11%) |
|  | *Significantly* | 80 (51%) |
|  | *Neutral* | 26 (17%) |
|  | *Slightly* | 30 (19%) |
|  | *Not at all* | 3 (2%) |
| **Do you believe that your fatigue during night-time work may increase the perioperative risk of your patients?** | *Very much* | 21 (13%) |
|  | *To some degree* | 82 (52%) |
|  | *Neutral* | 28 (18%) |
|  | *Rarely* | 17 (11%) |
|  | *Not at all* | 9 (6%) |
| **Taking into account your current work conditions, please rate your opinion on the following sentence: 'Night-time work represents an additional risk per se for the patient'** | *Strongly agree* | 22 (14%) |
|  | *Agree* | 91 (58%) |
|  | *Neutral* | 30 (19%) |
|  | *Disagree* | 12 (8%) |
|  | *Strongly disagree* | 2 (1%) |
| **Do you believe that the overall working conditions at your hospital during night-time may increase the perioperative risk of your patients?** | *Very much* | 30 (19%) |
|  | *Partly* | 86 (55%) |
|  | *Neutral* | 19 (12%) |
|  | *Rarely* | 17 (11%) |
|  | *Not at all* | 5 (3%) |

Data are reported as number and percentages.

**Table S5. Demographic characteristics of the respondents declaring nightwork affects, significantly or extremely, their quality of daily life.**

|  | | | All  (n=840) | |
| --- | --- | --- | --- | --- |
|  | | | N (%) | |
| **Please indicate your age** | *< 30 years old* | 50 (6%) | |  |
|  | *> 55 years old* | 129 (15%) | |  |
|  | *30-40 years old* | 342 (41%) | |  |
|  | *41-55 years old* | 319 (38%) | |  |
| **Please indicate your status** | *Junior consultant (<10 years since passing boards)* | 306 (36%) | |  |
|  | *Senior consultant* | 432 (52%) | |  |
|  | *Trainee* | 102 (12%) | |  |
| **Please indicate to which gender identity do you most identify?** | *Female* | 496 (59%) | |  |
|  | *Male* | 344 (41%) | |  |
| **Is the hospital where you work:** | *Academic* | 381 (45%) | |  |
|  | *Non academic* | 459 (55%) | |  |
| **Is the hospital where you work:** | *Private* | 61 (7%) | |  |
|  | *Public* | 779 (93%) | |  |

Data are reported as number and percentages.

**Table S6. Nighttime workload characteristics of the respondents declaring nightwork affects, significantly or extremely, their quality of daily life.**

|  | | All  (n=840) |
| --- | --- | --- |
|  | | N (%) |
| **How many nights are you on-call (standby at home) every month?** | | 3 [1-4] |
| **On nights that you are on call (standby at home), how many times you get called into the hospital on an average?** | *I’m not on call (at home)* | 184 (22%) |
|  | *Almost every time* | 111 (13%) |
|  | *Occasionally* | 316 (38%) |
|  | *Rarely* | 229 (27%) |
| ***How many nights do you work on site (all night in the hospital) every month?*** | | 4 [4-5] |
| **How many times are you called per night when you are on site shift (all night in the in-hospital)?** | *I’m not on site* | 13 (1%) |
|  | *2-4 times* | 324 (39%) |
|  | *Five times or more* | 214 (25%) |
|  | *One time a night at most* | 40 (5%) |
|  | *We work all night running* | 249 (30%) |
| **When you work during the night, how many consecutive work hours does your shift include overall?** | *I’m not on site* | 13 (1%) |
|  | *6* | 86 (10%) |
|  | *8* | 67 (8%) |
|  | *12* | 628 (75%) |
|  | *16* | 21 (3%) |
|  | *24* | 15 (2%) |
|  | *Other* | 10 (1%) |
| **Which kind of schedule strategy is employed in your hospital?** | *Consecutive night shifts (number of night shifts in a row alternating with number of day shifts in a row)* | 21 (3%) |
|  | *Irregular night shifts* | 603 (71%) |
|  | *Permanent night shift work (primarily or only night shifts)* | 30 (4%) |
|  | *Rotating shift with 1 day off (night shift and day shift together and then 1 recovery day)* | 150 (18%) |
|  | *Rotating shift with 2 days off (night shift and day shift together and then 2 recovery days)* | 36 (4%) |
| **Is it common for you to work at night after having worked on the same day?** | *No, never* | 413 (49%) |
|  | *Rarely* | 223 (27%) |
|  | *Sometimes* | 111 (13%) |
|  | *Yes, frequently* | 60 (7%) |
|  | *Yes, always* | 33 (4%) |
| **What type of patient do you attend to during night-time?** | *Post Anesthesia Care Unit / Intensive Care Unit team activity* | 526 (63%) |
|  | *Emergency surgery* | 623 (74%) |
|  | *Elective Surgery* | 83 (10%) |
|  | *Emergency medical team activity* | 414 (49%) |

Data are reported as number and percentages.

**Table S7. Results of questions on facilities and advocacy of the respondents declaring nightwork affects, significantly or extremely, their quality of daily life.**

|  | | All  (n=840) |
| --- | --- | --- |
|  | | N (%) |
| **Does your hospital have rooms dedicated to relaxation for all doctors working at night?** | *No* | 228 (27%) |
|  | *Yes* | 612 (73%) |
| **Does your hospital have rest facilities available for doctors who have worked during the night, to be used before returning home?** | *No* | 703 (84%) |
|  | *Yes* | 137 (16%) |
| **Does your hospital provide free meals, snacks, and beverages (i.e. water, coffee, tea) to doctors working at night?** | *No* | 754 (90%) |
|  | *Yes* | 86 (10%) |
| **Were you informed of the consequences of nightwork before you started working as a trainee?** | *No* | 700 (83%) |
|  | *Yes* | 140 (17%) |
| **Have you ever received training, information or tips on how to improve your performance when working at night?** | *No* | 813 (97%) |
|  | *Yes* | 27 (3%) |
| **Does your hospital have a program to monitor stress or fatigue in night shift workers?** | *No* | 833 (99%) |
|  | *Yes* | 7 (1%) |
| **During night-time, do you have the possibility to discuss clinical issues or involve another colleague in difficult clinical decisions?** | *Usually, no* | 161 (19%) |
|  | *Sometimes* | 332 (40%) |
|  | *Yes, always when I feel I need it* | 347 (41%) |
| **During night-time, do you feel comfortable calling the person 'on call' to come into the hospital?** | *No* | 311 (37%) |
|  | *Sometimes* | 263 (31%) |
|  | *Yes* | 266 (32%) |
| **You responded 'only sometimes' or 'no' please state the MAIN cause:**  **(Total=574)** | *Other* | 25 (4%) |
|  | *The person I am calling in is senior to me* | 43 (8%) |
|  | *The person I am calling in may argue with me about the need to come on the basis of case complexity or the number of cases* | 127 (22%) |
|  | *The person I am calling in or my colleagues may think I am not suited for this job* | 21 (4%) |
|  | *The person I am calling in will be calling me in on another day.* | 34 (6%) |
|  | *The person I am calling in will be judging my performance when my tenure/promotion is brought up for discussion* | 18 (3%) |
|  | *The person I am calling works on the following day and I do not want to deprive them of sleep.* | 306 (53%) |

Data are reported as number and percentages.

**Table S8. Results of questions on patients’ safety and doctors’ quality of life of the respondents declaring nightwork affects, significantly or extremely, their quality of daily life.**

|  |  | All  (n=840) |
| --- | --- | --- |
|  |  | N (%) |
| **Please indicate how much you think your nightwork affects the quality of your daily life** | *Extremely* | 161 (19%) |
|  | *Significantly* | 679 (81%) |
|  | *Neutral* | 0 (0%) |
|  | *Slightly* | 0 (0%) |
|  | *Not at all* | 0 (0%) |
| **Do you believe that sleep deprivation affects your professional performance?** | *Extremely* | 135 (16%) |
|  | *Significantly* | 506 (60%) |
|  | *Neutral* | 115 (14%) |
|  | *Slightly* | 73 (9%) |
|  | *Not at all* | 11 (1%) |
| **Do you believe that your fatigue during night-time work may increase the perioperative risk of your patients?** | *Very much* | 152 (18%) |
|  | *To some degree* | 431 (51%) |
|  | *Neutral* | 123 (15%) |
|  | *Rarely* | 104 (12%) |
|  | *Not at all* | 30 (4%) |
| **Taking into account your current work conditions, please rate your opinion on the following sentence: 'Night-time work represents an additional risk per se for the patient'** | *Strongly agree* | 218 (26%) |
|  | *Agree* | 473 (56%) |
|  | *Neutral* | 112 (13%) |
|  | *Disagree* | 31 (4%) |
|  | *Strongly disagree* | 6 (1%) |
| **Do you believe that the overall working conditions at your hospital during night-time may increase the perioperative risk of your patients?** | *Very much* | 207 (25%) |
|  | *Partly* | 460 (55%) |
|  | *Neutral* | 95 (11%) |
|  | *Rarely* | 59 (7%) |
|  | *Not at all* | 19 (2%) |

Data are reported as number and percentages.

**Table S9. Demographic characteristics of the respondents declaring being called at work almost every time when on-call.**

|  | | | All  (n=130) | |
| --- | --- | --- | --- | --- |
|  | | | N (%) | |
| **Please indicate your age** | *< 30 years old* | 3 (2%) | |  |
|  | *> 55 years old* | 22 (17%) | |  |
|  | *30-40 years old* | 62 (48%) | |  |
|  | *41-55 years old* | 43 (33%) | |  |
| **Please indicate your status** | *Junior consultant (<10 years since passing boards)* | 50 (38%) | |  |
|  | *Senior consultant* | 71 (55%) | |  |
|  | *Trainee* | 9 (7%) | |  |
| **Please indicate to which gender identity do you most identify?** | *Female* | 70 (54%) | |  |
|  | *Male* | 60 (46%) | |  |
| **Is the hospital where you work:** | *Academic* | 62 (48%) | |  |
|  | *Non academic* | 68 (52%) | |  |
| **Is the hospital where you work:** | *Private* | 7 (5%) | |  |
|  | *Public* | 123 (95%) | |  |

Data are reported as number and percentages.

**Table S10. Nighttime workload characteristics of the respondents declaring being called at work almost every time when on-call.**

|  | | All  (n=130) |
| --- | --- | --- |
|  | | N (%) |
| **How many nights are you on-call (standby at home) every month?** | | 4 [3-6] |
| **On nights that you are on call (standby at home), how many times you get called into the hospital on an average?** | *I’m not on call (at home)* | 0 (%) |
|  | *Almost every time* | 130 (100%) |
|  | *Occasionally* | 0 (%) |
|  | *Rarely* | 0 (%) |
| ***How many nights do you work on site (all night in the hospital) every month?*** | | 4 [3-5] |
| **How many times are you called per night when you are on site shift (all night in the in-hospital)?** | *I’m not on site* | 4 (3%) |
|  | *2-4 times* | 34 (26%) |
|  | *Five times or more* | 29 (23%) |
|  | *One time a night at most* | 7 (5%) |
|  | *We work all night running* | 56 (43%) |
| **When you work during the night, how many consecutive work hours does your shift include overall?** | *I’m not on site* | 4 (3%) |
|  | *6* | 10 (8%) |
|  | *8* | 9 (7%) |
|  | *12* | 98 (75%) |
|  | *16* | 6 (4%) |
|  | *24* | 2 (2%) |
|  | *Other* | 1 (1%) |
| **Which kind of schedule strategy is employed in your hospital?** | *Consecutive night shifts (number of night shifts in a row alternating with number of day shifts in a row)* | 5 (4%) |
|  | *Irregular night shifts* | 88 (67%) |
|  | *Permanent night shift work (primarily or only night shifts)* | 6 (5%) |
|  | *Rotating shift with 1 day off (night shift and day shift together and then 1 recovery day)* | 25 (19%) |
|  | *Rotating shift with 2 days off (night shift and day shift together and then 2 recovery days)* | 6 (5%) |
| **Is it common for you to work at night after having worked on the same day?** | *No, never* | 35 (27%) |
|  | *Rarely* | 29 (22%) |
|  | *Sometimes* | 28 (22%) |
|  | *Yes, frequently* | 25 (19%) |
|  | *Yes, always* | 13 (10%) |
| **What type of patient do you attend to during night-time?** | *Post Anesthesia Care Unit / Intensive Care Unit team activity* | 82 (63%) |
|  | *Emergency surgery* | 108 (83%) |
|  | *Elective Surgery* | 25 (19%) |
|  | *Emergency medical team activity* | 55 (42%) |

Data are reported as number and percentages.

**Table S11. Results of questions on facilities and advocacy of the respondents declaring being called at work almost every time when on-call.**

|  | | All  (n=130) |
| --- | --- | --- |
|  | | N (%) |
| **Does your hospital have rooms dedicated to relaxation for all doctors working at night?** | *No* | 43 (33%) |
|  | *Yes* | 87 (67%) |
| **Does your hospital have rest facilities available for doctors who have worked during the night, to be used before returning home?** | *No* | 109 (84%) |
|  | *Yes* | 21 (16%) |
| **Does your hospital provide free meals, snacks, and beverages (i.e. water, coffee, tea) to doctors working at night?** | *No* | 112 (86%) |
|  | *Yes* | 18 (14%) |
| **Were you informed of the consequences of nightwork before you started working as a trainee?** | *No* | 98 (75%) |
|  | *Yes* | 32 (25%) |
| **Have you ever received training, information or tips on how to improve your performance when working at night?** | *No* | 125 (96%) |
|  | *Yes* | 5 (4%) |
| **Does your hospital have a program to monitor stress or fatigue in night shift workers?** | *No* | 130 (100%) |
|  | *Yes* | 0 (0%) |
| **During night-time, do you have the possibility to discuss clinical issues or involve another colleague in difficult clinical decisions?** | *Usually, no* | 28 (21%) |
|  | *Sometimes* | 54 (42%) |
|  | *Yes, always when I feel I need it* | 48 (37%) |
| **During night-time, do you feel comfortable calling the person 'on call' to come into the hospital?** | *No* | 45 (35%) |
|  | *Sometimes* | 41 (31%) |
|  | *Yes* | 44 (34%) |
| **You responded 'only sometimes' or 'no' please state the MAIN cause:**  **(Total=86)** | *Other* | 4 (5%) |
|  | *The person I am calling in is senior to me* | 8 (9%) |
|  | *The person I am calling in may argue with me about the need to come on the basis of case complexity or the number of cases* | 12 (14%) |
|  | *The person I am calling in or my colleagues may think I am not suited for this job* | 5 (6%) |
|  | *The person I am calling in will be calling me in on another day.* | 8 (9%) |
|  | *The person I am calling in will be judging my performance when my tenure/promotion is brought up for discussion* | 1 (1%) |
|  | *The person I am calling works on the following day and I do not want to deprive them of sleep.* | 48 (56%) |

Data are reported as number and percentages.

**Table S12. Results of questions on patients’ safety and doctors’ quality of life of the respondents declaring being called at work almost every time when on-call.**

|  |  | All  (n=130) |
| --- | --- | --- |
|  |  | N (%) |
| **Please indicate how much you think your nightwork affects the quality of your daily life** | *Extremely* | 24 (18%) |
|  | *Significantly* | 87 (67%) |
|  | *Neutral* | 14 (11%) |
|  | *Slightly* | 5 (4%) |
|  | *Not at all* | 0 (0%) |
| **Do you believe that sleep deprivation affects your professional performance?** | *Extremely* | 26 (20%) |
|  | *Significantly* | 61 (47%) |
|  | *Neutral* | 17 (13%) |
|  | *Slightly* | 21 (16%) |
|  | *Not at all* | 5 (4%) |
| **Do you believe that your fatigue during night-time work may increase the perioperative risk of your patients?** | *Very much* | 32 (25%) |
|  | *To some degree* | 62 (48%) |
|  | *Neutral* | 13 (10%) |
|  | *Rarely* | 16 (12%) |
|  | *Not at all* | 7 (5%) |
| **Taking into account your current work conditions, please rate your opinion on the following sentence: 'Night-time work represents an additional risk per se for the patient'** | *Strongly agree* | 38 (29%) |
|  | *Agree* | 75 (58%) |
|  | *Neutral* | 10 (8%) |
|  | *Disagree* | 7 (5%) |
|  | *Strongly disagree* | 0 (0%) |
| **Do you believe that the overall working conditions at your hospital during night-time may increase the perioperative risk of your patients?** | *Very much* | 36 (28%) |
|  | *Partly* | 65 (50%) |
|  | *Neutral* | 13 (10%) |
|  | *Rarely* | 12 (9%) |
|  | *Not at all* | 4 (3%) |

Data are reported as number and percentages.
